# *BCL11A* Facilitates Cell Proliferation and Metastasis in Neuroblastoma *via* Regulating the PI3K/Akt Signaling Pathway

**DOI:** 10.2174/1568009622666220728123748

**Published:** 2022-10-14

**Authors:** Qianya Jin, Yanmin Chen, Shibei Du, Dongqing Xu, Juanqing Yue, Lei Cai, Xiaojun Yuan

**Affiliations:** 1 Department of Pediatric Hematology/Oncology, Xinhua Hospital Affiliated to Shanghai Jiao Tong University School of Medicine, Shanghai 200092, China;; 2 Department of Medicine, Quzhou College of Technology, Quzhou, Zhejiang Province 324000, China;; 3 Department of Pathology, Xinhua Hospital Affiliated to Shanghai Jiao Tong University School of Medicine, Shanghai 200092, China

**Keywords:** Neuroblastoma, *BCL11A*, prognosis, proliferation, invasion, metastasis

## Abstract

***Purpose*:** The study aims to access the value of B-cell lymphoma/leukemia 11A (*BCL11A*) in the prognosis of patients with neuroblastoma (NB) and to explore its role and possible mechanism in NB.

***Methods*:** Tumor specimens from 53 children with neuroblastoma were evaluated for the relationship between *BCL11A* expression level and prognosis of NB patients. Online datasets like *SEQC* and *Asgharzadeh* were analyzed to further check out the suppose.The role of *BCL11A* in the proliferation and migration of NB cells was studied by functional experiments such as CCK8, colony formation, flow cytometry, transwell and wound healing assay after knocking down *BCL11A* by small interfering RNA (siRNA) *in vitro*. The protein makers of the potential pathways were tested by western blot.

***Results*:** High expression of *BCL11A* in NB patients was closely correlated with high-risk and poor prognosis. The proliferation and migration abilities of NB cell lines SK-N-BE(2) and IMR-32 were significantly impaired by silencing *BCL11A*. Downregulation of BCL11A expression level in NB cells inhibited the epithelial-mesenchymal transition (EMT) process and affected the PI3K/Akt signaling pathway.

***Conclusion*:** As a prognostic indicator of survival in NB patients, *BCL11A* might serve as a potential therapeutic target. *BCL11A* played a regulatory role in cell proliferation, invasion, and migration in NB, which may be through the PI3K/AKT signaling pathway and induce EMT.

## 1 INTRODUCTION

Neuroblastoma (NB) is the most common extracranial solid tumor in early childhood, accounting for approximately 8% to 10% of all pediatric tumors and 15% of tumor-related deaths in children [1]. NB is an enigmatic tumor with high heterogeneity in clinical outcomes, with spontaneous regression among low-risk cases but relentless progression in high-risk ones. With the progress in the therapeutic area in the recent two decades, the survival of low- and intermediate-risk NB are improved substantially. However, the long-term survival rate among patients with high-risk NB is lower than 50% [2, 3]. Therefore, new therapeutic strategies for children with high-risk NB are urgently needed.

As a transcription factor, B-cell lymphoma/leukemia 11A (*BCL11A*) plays an essential role in normal development, such as the switch of hemoglobin, migration of projection neurons, and lymphopoiesis [4]. *BCL11A* was initially recognized in B-cell non-Hodgkin lymphoma (B-NHL) and Hodgkin’s disease (HD) [5]. Recently, *BCL11A* has attracted widespread attention as a prognostic marker and potential therapeutic target in malignant neoplasms. The aberrant expression of *BCL11A* has been reported in acute myeloid leukemia [6], natural killer/T-cell lymphoma [7], breast cancer [8], lung cancer [9], and so on. Its increased expression resulted in malignant proliferation and metastasis, induced epithelial-mesenchymal transition and anticancer drug resistance, and its high expression level is associated with poor prognosis [10].

Moreover, the expression level of *BCL11A* is significantly elevated in high-risk neuroblastoma [11]. As a target of miR-146a, *BCL11A* can promote SK-N-SH cell growth and inhibit apoptosis [12]. However, the biological functions and clinical significance of *BCL11A* in NB remain unexplored.

This study aimed to investigate the relationship between *BCL11A* expression level and the clinical features in NB patients, as well as to explore the potential effect of *BCL11A* as a prognostic marker for NB patients. What’s more, the biological role of *BCL11A* in neuroblastoma was checked by silencing the *BCL11A* expression in neuroblastoma cell lines to explore its mechanism of tumor progression in NB.

## 2 MATERIALS AND METHODS

### 2.1 Clinical Samples and Cell Culture

Patients diagnosed with neuroblastoma in Xin Hua Hospital, affiliated with Shanghai Jiao Tong University School of Medicine, in the period of Sep. 2016 to July 2019, were enrolled to collect clinical information and tumor samples for further investigation. All patients were treated according to the Chinese Children Cancer Group-NB-2014 (CCCG-NB-2014) protocol [13]. This study was approved by the Ethics Committee of Xin Hua Hospital affiliated with Shanghai Jiao Tong University School of Medicine. The cell lines SK-N-SH, IMR-32, SH-SY5Y, and 293T were obtained from the Type Culture Collection of the Chinese Academy of Sciences (Shanghai, China). SK-N-BE(2) and SK-N-AS were purchased from ATCC (Manassas, USA). SK-N-SH, IMR-32, SK-N-BE(2), and 293T were all cultured in DMEM supplemented with 10% fetal bovine serum(FBS) and 1% penicillin-streptomycin solution. SH-SY5Y and SK-N-AS cell lines were cultured in a 1:1 mixture of MEM and F12 Medium with 1% Gluta-max, 1% Sodium pyruvate, 1% NEAA, 1% penicillin-streptomycin solution, and 10% FBS. The mediums and FBS were all purchased from Gibco, USA. All cells grew in a humidified incubator with 5% CO2 at 37°C.

### 2.2 *BCL11A* Expression Analysis in Human Cancer and NB Cell Lines

The *BCL11A* mRNA levels in different human cancers were analyzed by the Tumor Immune Estimation Resource (TIMER) database. The PrognoScan database (http://www.prognoscan.org/) was used to study the relationship between *BCL11A* expression and the prognosis of cancer patients. *BCL11A* expressions in normal tissues, tumors, and cell line datasets were visualized with the R2 genomics analysis and visualization platform (http://r2.amc.nl/). Additionally, the NB datasets *SEQC* and *Asgharzadeh* with information on clinical and prognostic factors were also available at the R2 genomics analysis and visualization platform.

### 2.3 Western Blot

Cells were lysed in the RIPA buffer (Beyotime) containing 1% PMSF (Beyotime). Protein concentrations were determined by the BCA assay (Beyotime). Equalized amounts of protein (20ug) were loaded and separated by SDS-PAGE and transferred to the PVDF membrane. After blocked, the membranes were incubated with the antibody and with an appropriate secondary antibody. The primary antibodies included N-cadherin (13116, 1:1000), E-cadherin (3195, 1:1000), Vimentin (5741, 1:1000), Slug (9585, 1:1000), Akt (4691, 1:1000), Phospho-Akt (4060, 1:1000), and β-actin (3700, 1:1000) from Cell Signaling Technology and *BCL11A* (19487, 1:1000) from Abcam.

### 2.4 Real-time PCR

According to the manufacturer’s instructions, total RNAs from cell lines were prepared through the miniBEST Universal RNA Extraction Kit (Takara). The mRNA of *BCL11A* was measured by SYBR Green-based RT-PCR (Takara) and determined by the 2^-ΔΔCT^ method. The following primers were used in this study: *BCL11A* forward: 5′-CCCCA-GCACTTAAGCAAACG-3′ and reverse: 5′-GTGGTCTGG-TTCATCATCTGTAAGA-3′. GAPDH forward: 5′-GGA-AGCTTGTCATCAATGGAAATC-3′ and reverse: 5′- TGA-TGACCCTTTTGGCTCCC-3′. The relative mRNA levels were calculated by comparing GAPDH in the same sample.

### 2.5 Immunohistochemical (IHC) Staining and Semi‐quantitative Analysis

After dewaxed, rehydrated, and processed for antigen retrieval, the paraffin-embedded sections were quenched with 3% H_2_O_2_ and blocked with 3% BSA. The slide was incubated with the primary antibody anti-*BCL11A* (1:100, Abcam) and with an HRP-conjugated secondary antibody. Staining was visualized by incubation with DAB.

Stained slides were evaluated by 2 experienced pathologists. Based on the staining intensity and the positive rate, a semiquantitative integration method was used to determine the results. The staining intensity score was as follows: colorless = 0, light yellow = 1, yellow-brown = 2, and brown = 3. The percentage of positive cells was scored as follows: < 25% = 1, 26% - 50% = 2, 51% - 75%= 3, >75% = 4. The result of staining was determined using the following formula: score = percentage score × staining intensity score [14, 15]. Median expression of BCL11A was used as the cutoff and patients were divided into high (n=27) and low (n=26) expression groups.

### 2.6 Cell Transfection

Neuroblastoma cells were transfected by the Lipofectin-mediated transfection method. The siRNA specific for *BCL11A* was synthesized (RiboBio) and the sequence was GGCAGCCTCTGCTTAGAAA. The control sequence was CAACAGGAGAGACCTTTAT. Cells were plated in six-well plates 24 h prior to transfection. According to the manufacturer’s instructions (www.thermofisher.cn/), the cell lines were cultured with the transfection complex (concluded 200ul Opti-MEM I, 6ul siRNA, 8ul Lipofectamine RNAiMAX, and complete medium) when the cell density reached 30-40%. After 24 h, the medium was changed to a complete medium. When cells reached 70-80% confluency, siRNA expressing cells were selected using a growth medium with 2 μg/mL puromycin (Beyotime). The Lipofectamine RNAiMAX was purchased from Invitrogen and Opti-MEM I medium was from Gibco.

### 2.7 Cell Proliferation Assay

Cell proliferation assay was performed at 48h after transfection, including Cell Counting Kit-8 (CCK-8; Yeasen), colony formation assay, and flow cytometry. For CCK-8, control and si-*BCL11A* NB cells were seeded on 96-well plates with 5 × 10^3^ cells per well. And each group was set in five parallel holes. The cells were incubated with 10μL CCK8 solution and 90μl RPMI 1640 phenol-free red medium in each well. The optical density was measured after 2 hours. Cell proliferation was examined at 0, 24, 48, and 72 hours.

For colony formation assay, cells were seeded on 6-well plates (1.0 ×10^3^ cells per well) and cultured for approximately 10 days. After the formation of colonies, cells were stained with crystal violet and then counted through five randomly selected fields by microscope at ×40 magnification, under bright-field illumination.

Cell cycle distribution was determined by flow cytometry. Cells in the logarithmic growth phase were collected and fixed with 70% ethanol overnight at 4°C. On the next day, the centrifuged cells were stained with 500μl propidium iodide (PI) Triton X-100 solution in the dark for 30 minutes at 37^o^C. All experiments were performed with at least 3 replicates.

### 2.8 Cell Invasion and Migration Analysis

Tumor cell migration ability was assessed by wound healing assay. The cell lines were transfected with siRNA and incubated in 6-well plates until cell density reached 95%. The confluent monolayer of cells was scratched by a sterile pipette tip and then incubated with serum-free DMEM and observed at ×200 magnification for 5 specific visual fields at 0h and 24h of wound initiation. The scratch healing rate =scratch width at (0h ‐24h) / 0h × 100%.

Invasion ability was analyzed through a transwell assay. Transwell assay was conducted after 48 hours of transfection with siRNA. The upper surfaces of the polycarbonic membranes of the transwell chambers (Corning Costar) were coated with 1:6 DMEM diluted Matrigel (Invitrogen). The lower chambers were filled with 500μL of DMEM supplemented with 15% FBS. Cells (2×10^5^) in 200μL DMEM were seeded into the upper chambers. After 48h incubation, cells that had migrated on the lower surface of the filters were fixed in 4% paraformaldehyde and stained with crystal violet. The cells were counted in 5 randomly selected visual fields under the microscope at ×200 magnification. All experiments were also performed with at least 3 replicates.

### 2.9 Statistical Analysis

Data analysis was performed by SPSS software version 22.0. The relationship between *BCL11A* expression level and clinical features was analyzed using the χ2 test. The independent sample t-test was used for the two groups and the one-way Analysis of Variance (ANOVA) was used among multiple groups. The overall survival (OS) and progression-free survival (PFS) were evaluated by the Kaplan-Meier method. Statistical significance was defined as p < 0.05.

## 3 RESULTS

### 3.1 The mRNA Expression of *BCL11A* in Different Human Cancers

**Fig. (1):**
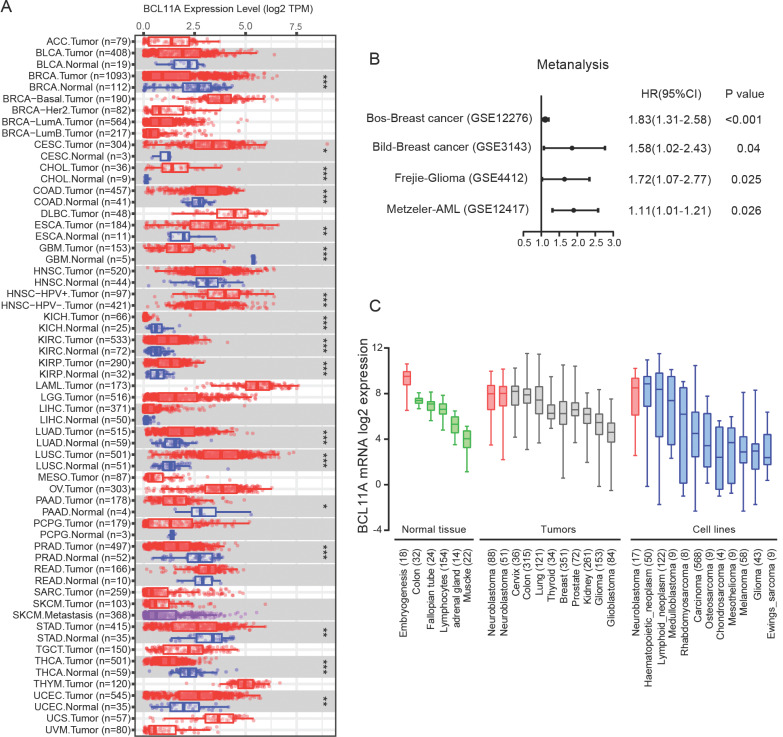
The expression of *BCL11A* in different human cancers. (**A**) *BCL11A* expression levels in different tumor types from the TCGA database were detected by TIMER. Compared with normal tissues, the expression level of BCL11A was higher in cholangiocarcinoma, colon adenocarcinoma, esophageal carcinoma, lung cancer and kidney cancer. *P < 0.05, **P < 0.01, ***P < 0.001 *vs*. normal tissues. (**B**) In 4 datasets, high expression of *BCL11A* was a risk factor for overall or disease‐free survival in patients with shorter OS or disease‐free survival (DFS) by PrognoScan database. (**C**) Compared to other cancers, high expression levels of *BCL11A* were observed in NB cell lines and primary tumors. *BCL11A* was highly expressed during embryogenesis.

The differences in *BCL11A* between various tumor tissues and the matched normal tissues were analyzed using previously published microarray gene expression datasets. Results showed that the expression level of *BCL11A* was higher in most cancers compared with normal tissues, such as cholangiocarcinoma, colon adenocarcinoma, esophageal carcinoma, lung cancer, kidney cancer, and so on (Fig. **1A**). The prognostic value of *BCL11A* in predicting patient outcomes was further investigated in the PrognoScan database. In the four datasets, high expression of *BCL11A* was related to shorter OS or disease‐free survival (DFS) (Cox P value < 0.05) (Fig. **1B**).

Compared with other cancers, higher expression levels of *BCL11A* were observed in NB cell lines and primary tumors (Fig. **1C**). Moreover, the *BCL11A* expression level was high in normal embryonic tissues. The above results strongly implied that *BCL11A* might play a vital role in the onset of neuroblastoma.

### 3.2 Elevated Expression of *BCL11A* was Correlated with Lower Survival in NB Patients

To explore the correlation between *BCL11A* expression and clinical outcomes of NB patients, clinical data and tumor tissues were studied in 53 patients. The patients consisted of 31 boys and 22 girls. Median age at diagnosis was 28 months. Of all 53 patients, 32 cases were neuroblastoma (NB), 3 cases were Ganglioneuroblastoma nodular type (GNBn), 17 cases were Ganglioneuroblastoma intermediate (GNBi), and one case was ganglioneuroma (GN). According to the International Neuroblastoma Staging System (INSS), 9 cases were stage 1, 7 cases were stage 2, 8 cases were stage 3, 24 cases were stage 4, and 5 cases were stage 4s. Among the 53 subjects, 23 received preoperative chemotherapy.

**Fig. (2):**
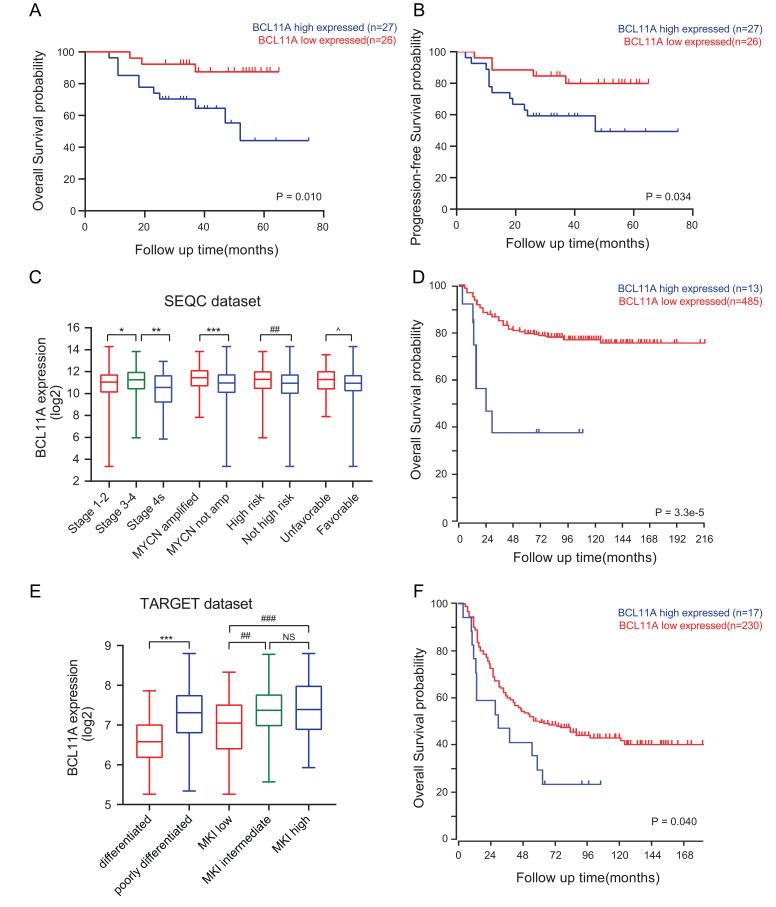
*BCL11A* was a potential prognostic marker in neuroblastoma. (**A**, **B**) Kaplan-Meier analysis of OS and PFS based on *BCL11A* expression (n = 53). (**C**) Differences between the expression level of *BCL11A* in neuroblastoma with different clinical characteristics were calculated by means of independent sample t-tests for the *SEQC* dataset. *P < 0.05 *vs.* stage 3-4, **P < 0.01 *vs.* stage 4s, ***P < 0.001 *vs. MYCN* not amplified. ^##^P < 0.01 *vs.* not high risk. ^p< 0.05 *vs.* favorable. (**D**) OS of the *SEQC* dataset based on *BCL11A* expression by Kaplan-Meier analysis. (**E**) For the *Asgharzadeh* dataset (n = 249), *BCL11A* was elevated in undifferentiated or poorly differentiated and MKI-high neuroblastoma tissues. ***P < 0.001 *vs.* undifferentiated or poorly differentiated. ^##^P < 0.01,^###^P < 0.001,^ns^P>0.05. The comparison was analyzed by t-test between two groups and by ANOVA among three groups. (**F**) OS of the *Asgharzadeh* dataset based on *BCL11A* expression by Kaplan-Meier analysis.

The staining of *BCL11A* in NB tissues was observed in the cytoplasm and nucleus of tumor cells. As shown in Fig. (**2A**), NB patients with high expression of *BCL11A* in tumor tissues had significantly poorer OS than those with low expression (*p*=0.010, Fig. **2A**). Among the 53 patients, 17 (32.1%) cases developed tumor progression or recurrence and 12 of them were defined as having high expression of *BCL11A*. Compared with the low-expression group, patients in high expression group were more likely to present with recurrence or progression (*p*=0.034, Fig. **2B**). Next, correlations between *BCL11A* and clinicopathological variables were analyzed, including gender, age, INSS stage, risk, *MYCN* status, pathological categories, and metastasis at diagnosis (Table **1**). Interestingly, the expressions of *BCL11A* were closely correlated with some indicators of NB progression, such as high-risk group (*P*=0.018). The above results suggested that *BCL11A* expression had a positive correlation with undesirable clinical characteristics in NB.

### 3.3 *BCL11A* was a Potential Prognostic Factor in Patients with NB

*SEQC* and *Asgharzadeh* dataset analysis provided further evidence of the correlation between *BCL11A* and the survival of NB patients. A total of 498 patients in the *SEQC* dataset and 249 patients in the *Asgharzadeh* dataset with complete follow-up information and gene expression profiles were recruited for survival analysis. In the *SEQC* dataset, patients with *MYCN* gene amplification and advanced stage NB were associated with a high expression of *BCL11A* (Fig. **2C**). Kaplan-Meier survival analysis showed that patients with high expression of *BCL11A* had a poorer prognosis in both datasets (*p*<0.05, Fig. **2D**, **2F**). In the Asgharzadeh dataset, high expression of *BCL11A* was significantly correlated with unfavorable histological subtypes such as undifferentiated and poorly differentiated NB and high mitosis karyorrhexis index (MKI) (Fig. **2E**). Taken together, high expression of *BCL11A* was related to high-risk and poor prognosis of neuroblastoma.

### 3.4 Estimation of Relative Expression of *BCL11A* in Six Cell Lines

**Fig. (3):**
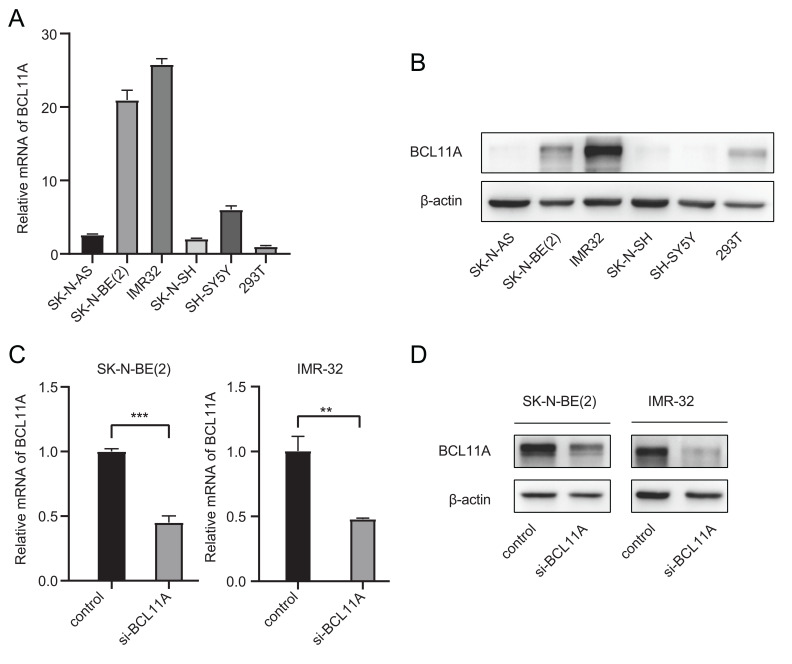
Expression of *BCL11A* in different cell lines and knockdown cell lines. (**A**, **B**) Using 293T cells as the control, the mRNA and protein expression levels of *BCL11A* in different neuroblastoma cell lines were assessed. (**C**, **D**) The transcription level and protein expression of *BCL11A* were decreased after transfected with si-*BCL11A*. **P < 0.01 and ***P < 0.001 *vs*. control.

To explore the effect of the *BCL11A* gene in neuroblastoma, cell culture experiments were used to provide more convincing evidence. Using 293T cells as control, the transcription level and protein expression of *BCL11A* were investigated by RT-PCR and Western blot in SK-N-AS, SK-N-BE(2), IMR-32, SK-N-SH, and SH-SY5Y. Among the five NB cell lines, IMR-32 and SK-N-BE(2) possessed high expression of *BCL11A* in mRNA and protein levels (Fig. **3A**, **B**).

Transient transfections were performed in SK-N-BE(2) and IMR-32 cell lines using the procedures described above. After interfered siRNA in the two cell lines, the mRNA and protein expressions of *BCL11A* were significantly decreased (Fig. **3C, D**).

### 3.5 Impaired Expression of *BCL11A* Inhibited Cell Proliferation

**Fig. (4):**
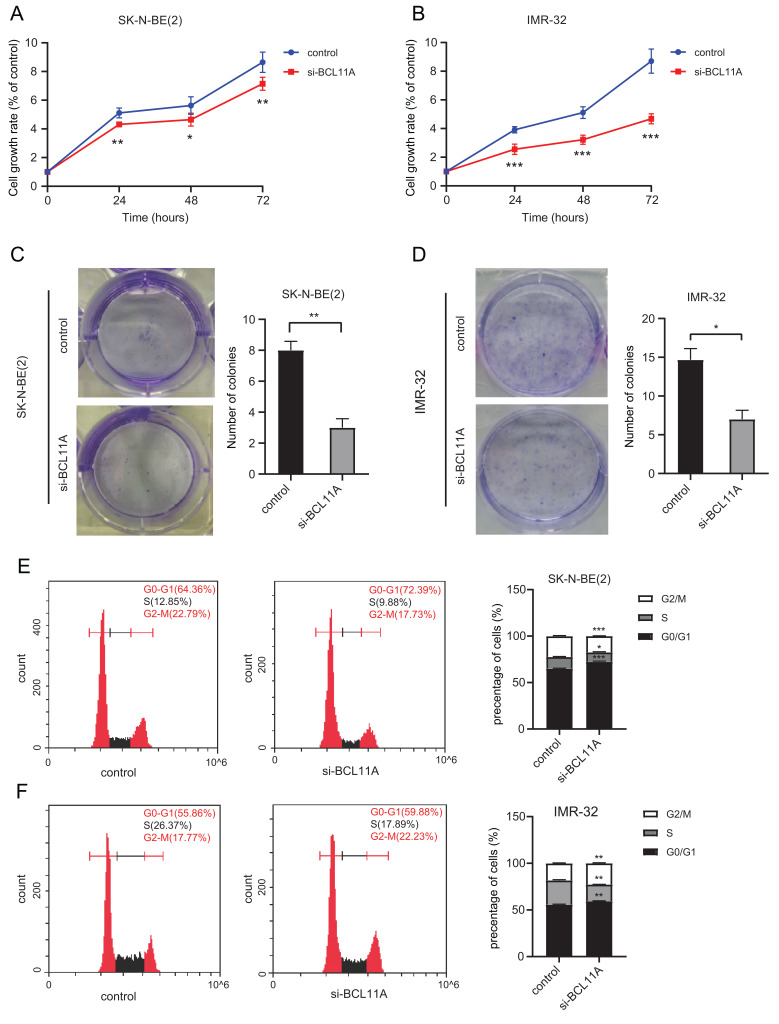
Knockdown of *BCL11A* inhibited cell proliferation in SK-N-BE(2) and IMR-32. (**A**, **B**) Cell growth of controlled and si-*BCL11A* cells was determined by CCK-8 assay. Data represent the mean ± SD, n=5. *P < 0.05, **P < 0.01, ***P < 0.001 *vs*. control. (**C**, **D**) Cell proliferation of controlled and si-*BCL11A* cells was examined by colony formation assay. *P < 0.05 and **P < 0.01 *vs*. control. (**E**, **F**) Cell cycle analysis was determined by flow cytometry in controlled and si-*BCL11A* cells. *P < 0.05, **P < 0.01, ***P < 0.001 *vs*. control. All experiments were repeated at least three times.

SK-N-BE(2) and IMR-32 cell growth were monitored *via* CCK8 assays for 3 consecutive days after siRNA transfection. Knockdown of *BCL11A* could significantly inhibit the proliferation in both IMR-32 and SK-N-BE(2) cell lines (Fig. **4A, B**). What’s more, colony formation assays demonstrated that, compared with control cells, knockdown of *BCL11A* remarkably suppressed the colony-forming ability (Fig. **4C, D**).

To further explore the role of *BCL11A* on the NB cell proliferation stage, flow cytometry was performed to analyze cell cycle arrest. As shown in Figs. (**4E**, **4F**), treatment with siRNA in SK-N-BE(2) and IMR-32 cells resulted in an increased accumulation of cell populations in the G0/G1 phase. These results indicated that *BCL11A* might promote NB cell proliferation.

### 3.6 Knockdown of *BCL11A* Suppressed Cell Invasion and Metastasis

**Fig. (5):**
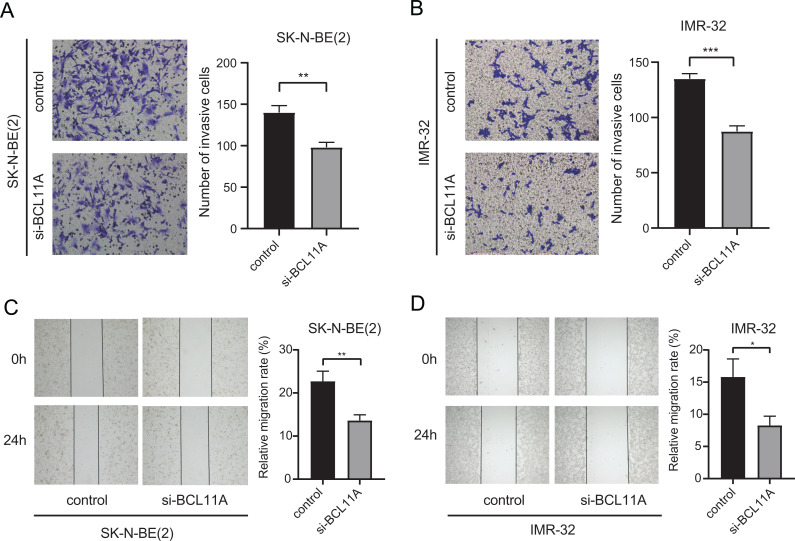
Knockdown of *BCL11A* suppressed cell metastasis in SK-N-BE(2) and IMR-32. (**A**, **B**) The ability of cell invasion in controlled and si-*BCL11A* cells by Transwell assay. **P < 0.01 and ***P < 0.001 *vs*. control. (**C**, **D**) Wound healing assay compared the migration in controlled and si-*BCL11A* cells. *P < 0.05 and **P < 0.01 *vs*. control. All experiments were repeated at least three times.

Transwell invasion assay demonstrated that NB cell lines with *BCL11A*-knockdown remarkably reduced the invasive capacity (Fig. **5A**, **B**). Meanwhile, the wound healing assay revealed that knockdown of *BCL11A* significantly inhibited cell migration in SK-N-BE(2) and IMR-32 (Fig. **5C**, **D**). These results demonstrated that the expression of *BCL11A* was related to the ability of tumor migration and invasion, which may be related to tumor metastasis.

### 3.7 *BCL11A* Might Promote EMT *via* Regulating the PI3K/Akt Signaling Pathway

**Fig. (6):**
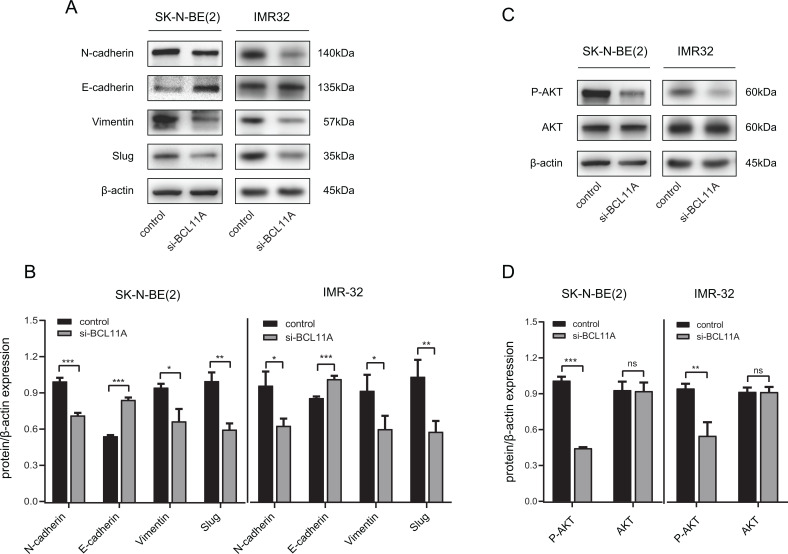
*BCL11A* promoted epithelial-mesenchymal transition (EMT) in NB cells. (**A**, **B**) Western blot of EMT-associated markers expression (epithelial marker E-cadherin, mesenchymal markers N-cadherin, vimentin, and Slug) in NB cell lines when *BCL11A* was downregulated. *P < 0.05, **P < 0.01, ***P < 0.001 *vs*. control. (**C, D**) Western blot of total Akt and phosphorylated Akt in control and si-*BCL11A* NB cells. **P < 0.01, ***P < 0.001,^ns^*P*>0.05 *vs*. control.

EMT is a critical process for cancer metastasis. To examine the influence of *BCL11A* expression on EMT in NB, the expression of epithelial and mesenchymal markers was measured by western blot. The results indicated that the expression level of the epithelial marker E-cadherin increased, whereas the mesenchymal markers such as N-cadherin, vimentin, and Slug decreased in si-*BCL11A* NB cells (Fig. **6A**, **B**). Therefore, we speculated that *BCL11A* regulated EMT in NB cells through EMT-associated transcription factors. According to past studies, EMT was regulated through several signaling pathways, including PI3K/AKT and Wnt/β-catenin [8, 13]. As shown in Figs. (**6C** and **6D**), the protein of phosphorylated Akt remarkably decreased in si-*BCL11A* NB cells, whereas there was no impact on total Akt protein expression. These results gave us a conjecture that *BCL11A* may induce EMT in NB cells through the PI3K/AKT signaling pathway, but more evidence was needed to confirm it.

## 4 DISCUSSION

Therefore far, mount studies have demonstrated that the overexpression of *BCL11A* in many tumors was correlated with poor prognosis [9, 16-19]. Consistent with these findings, our study identified that the prognosis of patients with high *BCL11A* expression was worse than those with low expression in neuroblastoma. Follow-up data also showed higher frequencies of recurrence or progression in tumors with high *BCL11A* expression. Compared with the non-high-risk group, the expression levels of *BCL11A* in the high-risk group were remarkably higher, either in our NB patient cohort or in the validation cohort. Although there was a previous study suggesting the possible association of *BCL11A* expression with high-risk neuroblastoma by database mining [11], our study further demonstrated the prognostic value of *BCL11A* in NB patients for the first time.

By analyzing the correlation between gene expression and clinical characteristics in patients with malignant solid tumors, the expression of *BCL11A* was significantly confirmed to be positively correlated with some tumor metastases, such as breast cancer [20], liver cancer [21], laryngeal squamous cell carcinoma [22], lung squamous cell carcinoma [23], and so on. Studies found that *BCL11A* could participate in epithelial-mesenchymal transition and promote breast cancer metastasis by the Wnt/β-catenin signaling pathway [8]. To explore the function of *BCL11A* in tumor growth and metastasis, functional tests were conducted by knocking down *BCL11A* in two neuroblastoma cell lines. The results prompted that down regulated expression of *BCL11A* inhibited the proliferation and invasion of neuroblastoma *in vitro*, which was similar to the results in NK/T lymphoma and prostate cancer [7, 24]. However, in our clinical study, patients with high *BCL11A* expression did not have obvious distant metastasis (*p*=0.075). This may be due to an insufficient sample size or a short follow-up period. Because, notably, one case with high expression of *BCL11A* in our study presented recurrence in the fifth year after being considered clinically cured, which means that high expression of *BCL11A* is detrimental to long-term prognostic outcomes.

The current mechanism research in neuroblastoma was limited to the role of *BCL11A* in cell apoptosis [12]. This study provided new evidence of tumorigenesis of *BCL11A* by promoting neuroblastoma cell proliferation and metastasis. The activation of EMT is commonly reflected as a vested feature of malignancy. EMT was also reported to be associated with the migratory and invasive properties of human NB cells. Phosphatidylinositol-3-kinase (PI3K) is a key signaling molecule in many cell activities, which can regulate a series of biological processes such as cell division, differentiation, apoptosis and so on. Especially, there is increasing evidence that the PI3K/Akt pathway plays an important role in the development and progression of NB [25]. It may be considered a novel therapeutic strategy in NB [26, 27]. The PI3K/Akt signaling pathway can induce the EMT process and inhibit the transcription of E-cadherin. In our study, we found that downregulation of *BCL11A* depressed EMT program in NB cells, including upregulation of the epithelial marker E-cadherin, as well as downregulation of mesenchymal markers (N-cadherin, slug, and vimentin). Although the process of mesenchymal to epithelial transition mediated by downregulation of *BCL11A* was incomplete, changes in cell migration and invasion did occur.

*MYCN* is a member of the MYC family of proto-oncogenes encoding the transcription factor N-MYC. It is known for its onco-genetic role and mechanisms in the prognosis of neuroblastoma and is considered one of the prominent targets for NB therapy [28]. According to our results, the expression level of *BCL11A* in SK-N-BE(2) and IMR-32 was higher than in SK-N-AS, SK-N-SH and SH-SY5Y cell lines. Interestingly, it has been reported that SK‐N‐BE(2) and IMR-32 cell lines are known to have *MYCN* amplification, whereas the other three cell lines are not [29]. What’s more, in the *SEQC* dataset, patients with *MYCN* gene amplification were associated with a high expression of *BCL11A* (Fig. **2C**). Therefore, there might be crosstalk between *BCL11A* and *MYCN* involved pathways. This speculation requires further experimental to verify. Interestingly, the expression of *BCL11A* was different in gender, with higher *BCL11A* expression in boys with NB. It was previously reported that neuroblastoma was more common in boys than in girls, which may contribute to this discrepancy [2, 30]. Further research with larger sample sizes is warranted.

## CONCLUSION

In conclusion, this study clarified the high expression level of proto-oncogene *BCL11A* in neuroblastoma tumor tissue. Moreover, data from our case, along with data from online datasets, revealed that the high expression of *BCL11A* in NB patients was notably related to high-risk, distant metastasis, and poor prognosis. Functional experiments verified that *BCL11A* played a regulatory role in cell proliferation, invasion, and migration in NB. The potential mechanism of *BCL11A* in NB cells may induce EMT through the PI3K/AKT signaling pathway. Therefore, *BCL11A* may serve as a novel prognostic predictor and a promising target for NB therapy.

## Figures and Tables

**Table 1:** The correlation between *BCL11A* expression and clinical features in 53 children with NB.

**Characteristics**	**Number of Patients**	***BCL11A* Expression**		**p-Value**
		**Low**	**High**	
**Gender**				0.019
Male	31	11	20	
Female	22	15	7	
**Age**				0.854
<18 months	19	9	10	
≥18 months	34	17	27	
**Stage**				0.340
I,II, IVs	21	12	9	
III,IV	32	14	18	
**Risk Group**				0.018
Low, Intermediate	30	19	11	
High	23	7	16	
***MYCN* Status**				0.252†
Amplified	8	2	6	
Not Amplified	44	23	21	
Not available	1	1	0	
**Pathological Subtype**				0.066
NB,GNBn	35	14	21	
GNBi, GN	18	12	6	
**Distant Metastasis**				0.075
Yes	29	11	18	
No	24	15	9	

**Notes:** NB, neuroblastoma; GNBn, ganglioneuroblastoma, nodular type; GNBi, ganglioneuroblastoma, intermediate; GN, ganglioneuroma.

^†^Denotes the difference between *MYCN* amplified and not amplified.

## Data Availability

Data analyzed in this article were available in public databases (TIMER, PrognoScan, and R2). The results published here are partly based upon data generated by the Tumor Immune Estimation Resource (TIMER) (http://timer.cistrome.org/) and the R2 genomics analysis and visualization platform (http://r2.amc.nl/).

## LIST OF ABBREVIATIONS

*BCL11A*: B-cell Lymphoma/Leukemia 11A
NB: Neuroblastoma
EMT: Epithelial-Mesenchymal Transition
B-NHL: B-cell Non-Hodgkin’s Lymphoma
HD: Hodgkin’s Disease
