# Supplementary material for: BCL11A Facilitates Cell Proliferation and Metastasis in Neuroblastoma via Regulating the PI3K/Akt Signaling Pathway
Source: Curr Cancer Drug Targets. 2022 Oct 14;22(11):919–30. doi: 10.2174/1568009622666220728123748 (PMC9900700; doi:10.2174/1568009622666220728123748)
Supplement: Supplementary file 1 — Supplementary material is available on the publisher’s web site along with the published article. [file CCDT-22-919_SD1.pdf]

Supplementary Materials

BCL11A Facilitates Cell Proliferation and Metastasis in Neuroblastoma *via* Regulating the PI3K/Akt Signaling Pathway

Qianya Jin[1,3,#], Yanmin Chen[1,#], Shibei Du[1], Dongqing Xu[1], Juanqing Yue[2], Lei Cai[2], and Xiaojun Yuan[1,*]

[1]Department of Pediatric Hematology/Oncology, Xinhua Hospital Affiliated to Shanghai Jiao Tong University School of Medicine, Shanghai 200092, China; [2]Department of Pathology, Xinhua Hospital Affiliated to Shanghai Jiao Tong University School of Medicine, Shanghai 200092, China; [3]Department of Medicine, Quzhou College of Technology, Quzhou, Zhejiang Province, China

SUPPLEMENTARY TABLE S1

Patient No.	Age (m)	Sex	Stage	Distant Metastases	Risk	MYCN Status	Pathological Subtype	BCL11A Expression	Follow-Up for OS (m)	Out-come	Follow-Up for PFS (m)	Progression or Recurrence
1	49	F	II	Negative	Intermediate	Normal	GNBi	Low	65	Alive	65	No
2	66	M	II	Negative	Low	Normal	NB	High	64	Alive	64	No
3	8	F	II	Negative	Intermediate	Normal	NB	Low	62	Alive	62	No
4	19	M	IV	Postive	High	Normal	NB	Low	61	Alive	61	No
5	9	M	I	Negative	Low	Normal	NB	High	57	Alive	57	No
6	37	F	III	Negative	High	Amplified	NB	Low	57	Alive	57	No
7	51	F	IV	Postive	High	Normal	GNBi	Low	56	Alive	56	No
8	44	M	I	Negative	Low	Normal	GNBi	Low	55	Alive	55	No
9	50	F	II	Negative	Low	Normal	GNBi	Low	55	Alive	55	No
10	39	M	IV	Postive	High	Normal	GNBi	Low	19	Die	12	Yes
11	1	M	I	Negative	Low	Normal	NB	Low	55	Alive	55	No
12	52	F	IV	Postive	High	Normal	NB	High	52	Die	24	Yes
13	98	F	IV	Postive	High	Normal	NB	Low	54	Alive	26	Yes
14	28	F	IV	Postive	High	Normal	NB	High	18	Die	12	Yes
15	44	M	IV	Postive	High	Normal	GNBi	High	11	Die	10	Yes
16	4	M	IVs	Postive	Intermediate	Normal	NB	Low	53	Alive	53	No
17	37	F	III	Negative	Intermediate	Normal	GNBi	High	52	Alive	52	No
18	42	M	IV	Postive	High	Normal	NB	High	37	Die	23	Yes
19	3	F	IVs	Postive	Intermediate	Normal	GNBn	Low	50	Alive	50	No
20	55	M	IV	Postive	High	Normal	NB	High	25	Die	19	Yes
21	63	F	III	Negative	Intermediate	Normal	GNBi	Low	48	Alive	48	No
22	66	M	IV	Postive	High	Normal	GNBi	High	44	Alive	18	Yes
23	10	M	IV	Postive	High	Normal	NB	High	11	Die	11	Yes

(Table S1) contd…..

1873-5576/22 $65.00+.00 © 2022 Bentham Science Publishers

Patient No.	Age (m)	Sex	Stage	Distant Metastases	Risk	MYCN Status	Pathological Subtype	BCL11A Expression	Follow-Up for OS (m)	Out-come	Follow-Up for PFS (m)	Progression or Recurrence
24	30	M	III	Negative	Intermediate	Normal	GNBn	High	40	Alive	40	No
25	3	F	III	Negative	Intermediate	Normal	NB	Low	38	Alive	38	No
26	7	M	III	Negative	Intermediate	Normal	NB	Low	42	Alive	42	No
27	27	F	IV	Postive	High	Normal	GNBi	High	11	Die	11	Yes
28	2	M	IV	Postive	Intermediate	Normal	NB	Low	38	Alive	38	No
29	79	M	III	Negative	Intermediate	Normal	GNBi	Low	37	Alive	37	No
30	124	F	I	Negative	Low	Normal	GNBi	Low	35	Alive	35	No
31	11	M	I	Negative	Low	Normal	NB	High	34	Alive	34	No
32	24	M	IV	Postive	High	Amplified	NB	Low	15	Die	12	Yes
33	72	F	IV	Postive	High	Normal	NB	Low	33	Alive	6	Yes
34	4	M	I	Negative	Low	Normal	NB	High	33	Alive	33	No
35	17	M	II	Negative	Intermediate	Normal	NB	High	32	Alive	32	No
36	65	F	III	Negative	Intermediate	Normal	GNBi	Low	32	Alive	32	No
37	1	F	I	Negative	Low	Normal	NB	High	28	Alive	28	No
38	18	M	IV	Postive	High	Normal	NB	High	27	Alive	27	No
39	50	F	I	Negative	Low	Normal	GNBi	Low	27	Alive	27	No
40	40	M	IV	Postive	High	Normal	NB	High	26	Alive	26	No
41	20	F	IV	Postive	High	Amplified	GNBn	High	47	Die	47	Yes
42	124	M	II	Negative	Low	Normal	GNBi	Low	38	Alive	38	No
43	8	M	II	Negative	Low	Normal	NB	High	38	Alive	38	No
44	3	M	IVs	Postive	Intermediate	Normal	NB	Low	59	Alive	59	No
45	2	F	IV	Postive	Intermediate	Normal	NB	Low	37	Die	37	Yes
46	1	F	IVs	Postive	Intermediate	Normal	NB	High	41	Alive	41	No
47	2	M	IVs	Postive	Intermediate	Normal	NB	High	32	Alive	32	No
48	7	M	IV	Postive	High	Amplified	NB	High	49	Alive	49	No
49	32	M	IV	Postive	High	Amplified	GNBi	High	8	Die	3	Yes
50	56	M	IV	Postive	High	Amplified	GNBi	High	75	Alive	75	No
51	19	M	IV	Postive	High	Amplified	NB	High	18	Die	5	Yes
52	22	M	IV	Postive	High	Amplified	NB	High	23	Die	11	Yes
53	80	F	I	Negative	Low	Not available	GN	Low	34	Alive	34	No

Notes: NB, neuroblastoma; GNBn, ganglioneuroblastoma, nodular type; GNBi, ganglioneuroblastoma, intermediate; GN, ganglioneuroma.
